# Spontaneous pneumomediastinum mimicking acute pericarditis

**DOI:** 10.1002/ccr3.5156

**Published:** 2021-12-04

**Authors:** Haseeb Chaudhary, Zohaib Yousaf, Usama Nasir, Tayyab Waheed, Khezar Syed

**Affiliations:** ^1^ Department of Medicine Reading Hospital Tower Health System Reading Pennsylvania USA; ^2^ Department of Medicine Hamad General Hospital Doha Qatar

**Keywords:** acute pericarditis, ECG, pneumomediastinum, ST‐T wave changes

## Abstract

ST elevations on electrocardiogram (ECG) have a broad differential diagnosis that can vary from benign to more ominous pathologies. These include early repolarization, coronary vasospasm, acute pericarditis, ST‐elevation myocardial infarction, ventricular aneurysms, and dissecting aneurysm of the aorta reaching the pericardium. ST‐segment changes may also provide a clue to the presence of spontaneous pneumomediastinum (SPM). These ECG changes are seldom reported in literature. We describe two SPM cases with concomitant pneumopericardium that closely mimicked acute pericarditis with a deceptive clinical spectrum.

## Graphical abstract

The atypical clinical presentation of pneumomediastinum may produce ECG changes. The ECG changes of pneumomediastinum can mimic acute pericarditis. Early recognition and appropriate treatment can lead to improved clinical outcomes.

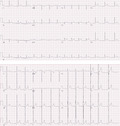

## 1 INTRODUCTION

ST elevations on ECG have a broad differential diagnosis that can vary from benign to more ominous pathologies. These include early repolarization, coronary vasospasm, acute pericarditis, ST‐elevation myocardial infarction, ventricular aneurysms, and dissecting aneurysm of the aorta reaching the pericardium.^1^, ^2^, ^3^, ^4^, ^5^ The presentation of these diseases can closely resemble SPM, in which patients often present with sudden onset chest pain and shortness of breath. Furthermore, the clinical scenario in patients with underlying SPM can pose a diagnostic challenge due to ST‐T changes on ECG that may mimic acute coronary syndrome.^6^, ^7^, ^8^ These ECG changes associated with SPM have seldom been reported in the literature. We describe two SPM cases with concomitant pneumopericardium that closely mimics acute pericarditis with a deceptive clinical spectrum.

## 2 CASE PRESENTATION

Patient characteristics are summarized in Table 1.

**TABLE 1:** Patient characteristics (ECG—electrocardiogram; CT—computed tomography, and LVH—left ventricular hypertrophy)

Patient characteristics	Case 1	Case 2
Age	19 years	19 years
Sex	Male	Male
Risk Factors	Occasional marijuana use, Smoking	Marijuana use, Vaping
Symptoms	Chest pain, shortness of breath, syncope	Hyperemesis, Chest pain
CRP	0.11 (<1.00 mg/dL)	0.50 (<1.00 mg/dL)
Troponin	0.03 (<0.06 ng/mL)	0.03 (<0.06 ng/mL)
ECG changes	ST‐T elevations V3‐V4 with PR elevation in AVR	Diffuse ST‐T elevations with PR elevation in AVR with evidence of LVH
Chest X‐ray	Subcutaneous emphysema, continuous diaphragm sign	The air along the mediastinum and subcutaneous emphysema
CT Findings	Subcutaneous emphysema, moderate pneumomediastinum with pneumopericardium, and a collapsed esophagus	Subcutaneous emphysema, moderate pneumomediastinum, pneumopericardium, esophagus not well visualized.
Echocardiogram	Normal function, no evidence of pericardial effusion.	Mild concentric left ventricular hypertrophy normal chamber size and hyperdynamic systolic function with no apparent regional wall motion abnormalities.
Esophagram	Not performed	No evidence of an esophageal leak

### 2.1 Case 1

**FIGURE 1:**
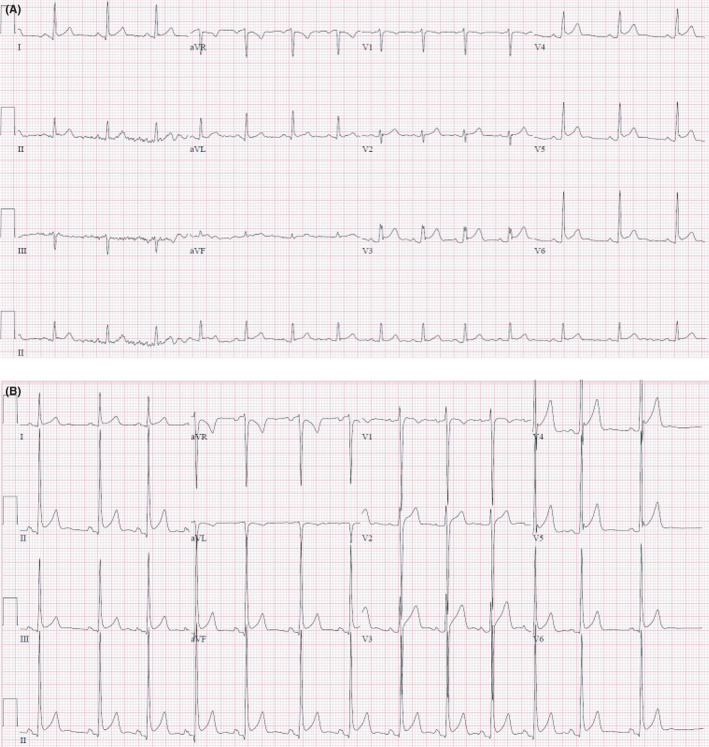
ECG demonstrating (A) ST‐T elevations V3‐V4 with PR elevation in AVR (B) Diffuse ST‐T elevations with PR elevation in AVR with evidence of LVH. (CT—computerized tomography and LVH—left ventricular hypertrophy)

**FIGURE 2:**
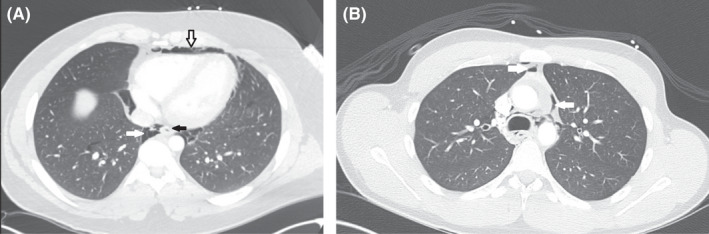
CT Thorax (Axial views) with (A) moderate pneumomediastinum with pneumopericardium (white arrows) and a collapsed esophagus (black arrow). (B) Moderate pneumomediastinum (white arrows), esophagus not well visualized

A 19‐year‐old gentleman with a history of occasional marijuana use presented with acute onset progressive central and sharp chest pain for several hours, followed by a brief syncopal episode. The pain was pleuritic and exacerbated on lying flat. He denied any trauma, fever, cough, retching, or vomiting. He did not have any previous history of hospitalizations. On presentation, the blood pressure was 117/79 mmHg with a pulse rate of 76 beats/min, temperature 37.7°C (99.9°F), and a respiratory rate of 22 breaths/min with an oxygen saturation of 96% on ambient air. He did not have orthostasis. There was crepitus around the left lower neck, appreciated on palpation, and precordial auscultation was significant for a Hamman's crunch best heard in the 4th left intercostal space suggestive of subcutaneous emphysema. Complete blood counts (CBC) revealed a white cell count (WBC) of 5.4 × 10^3^, hemoglobin 15 g/dL, and platelets 241 × 10^3^ per microliter. ECG showed ST‐segment elevations in the precordial leads (Figure 1A). Serial troponins were negative. Chest X‐ray revealed a continuous diaphragm sign suggestive of pneumomediastinum, which was confirmed by computed tomography (CT) of the chest showing evidence of spontaneous pneumomediastinum along with pneumopericardium (Figure 2A). The echocardiogram was unremarkable. An esophagram was not performed given the low suspicion for any esophageal rupture. The patient was successfully managed with mild analgesia, oxygen therapy, and clinical observation, with gradual resolution of ECG changes as the pneumopericardium resolved.

### 2.2 Case 2

A 19‐year‐old gentleman with a history of smoking and marijuana use presented with left‐sided chest pain, proceeded by intractable nausea and vomiting for 2 weeks. The pain radiated to the neck, upper back, and shoulders base and was not associated with shortness of breath. He did not report hematemesis or melena. He denied upper respiratory tract symptoms. On presentation, his blood pressure was 162/91 mmHg, pulse rate of 82/min, temperature 36.7°C (98.1°F), and oxygen saturation of 98% on ambient air. He had supraclavicular crepitus but otherwise normal systemic examination. CBC showed WBC count of 7.10 × 10^3^ per microliter, hemoglobin 13.8 g/dL, and platelet count of 214 × 10^3^ per microliter. ECG showed diffuse ST‐segment elevation, PR segment depression, and evidence of left ventricular hypertrophy, with no reciprocal changes suggestive of acute pericarditis (Figure 1B). Chest X‐ray showed pneumomediastinum. A subsequent CT chest showed air in the anterior mediastinum, pneumopericardium, and subcutaneous emphysema (Figure 2B). Esophagram did not show any evidence of a leak. The patient was initially started on ibuprofen and colchicine given the ECG changes highly suggestive of acute pericarditis, but later therapy was tailored to mild analgesia with clinical observation. ECG changes subsided upon resolution of pneumopericardium.

## 3 DISCUSSION

Pneumomediastinum relates to the accumulation of air in the mediastinal structures. It may result from blunt trauma leading to thoracic injury, esophageal perforation, or hollow viscus rupture. It can also occur spontaneously without any structural lung or mediastinal abnormalities in the absence of trauma, called SPM. In general, SPM can be caused by intrathoracic (involving trachea and major bronchial airways, esophagus, lung, and pleural cavity) or extra‐thoracic (head, neck, or peritoneum) processes. SPM is a non‐traumatic entity that occurs due to increased intrathoracic pressure leading to alveolar rupture. As the mediastinal pressure is more negative than the pulmonary parenchyma, the air enters the pulmonary interstitium. It dissects along the perivascular sheets to reach the hilum from where it spreads to the mediastinum. This phenomenon is described as the Macklin effect.^9^ Occasionally, pneumopericardium can also occur with leakage of air into the pericardial space.^10^ It is worth emphasizing, spontaneous alveolar rupture can occur even with no prior history of pulmonary disease or esophageal perforation and is usually precipitated by cough, emesis, physical exercise, labor, or upper airway infection. Breathing mechanics involved in marijuana smoking include inspiration and expiration against a closed airway (Muller's Maneuver and Valsalva maneuver, respectively), which predispose to barotrauma which is further exacerbated by hyperemesis and cough. Both our patients had SPM likely precipitated by cough and hyperemesis from marijuana use.^11^

The exact mechanism of ST‐segment changes secondary to SPM remains unclear. The most common ECG findings associated with SPM are the loss of R wave in the precordial leads and diminution of QRS voltages or ST‐segment elevation in inferior leads.^12^, ^13^, ^14^, ^15^, ^16^ Previously described mechanisms propose cardiac rotation, right ventricular dilatation, and insulation effect caused by air accumulation between the cardiac structures and the chest wall as the cause of such changes. Secondly, SPM may lead to myocardial stretch leading to stretching and narrowing of the coronary arteries, which may masquerade as ST‐elevation myocardial infarction with a troponin leak.^16^ We further postulate the ECG changes associated with pneumopericardium in our cases could be related to the direct inflammatory effect from air leakage between the pericardium and the chest wall leading to a presentation similar to acute pericarditis. Such ST‐T changes may give an initial diagnostic clue of the presence of pneumopericardium accompanying SPM. The concurrent pneumopericardium diagnosis may be relevant if caused by fistula formation from the adjacent intra‐abdominal structures as mortality rates can be as high as 50–70% in such cases.^17^, ^18^ Given the lack of abdominal symptoms and CT findings not suggestive of an intra‐abdominal or esophageal source in Case 1, an esophagram was not performed. The esophagram in the second described case was performed as the patient presented with hyperemesis, leading to a suspicion of Boerhaave syndrome. These patients appear sicker on presentation with hypotension and shock due to chemical mediastinitis, and a pleural effusion often accompanies findings of SPM.^19^

Management of SPM is mainly conservative with avoidance of trigger factors, analgesia, bed rest, and oxygen therapy in the absence of complications such as hemodynamic instability, pneumothorax, suspected chemical mediastinitis, or tamponade effect by the coexisting pneumopericardium.

## 4 CONCLUSION

Our cases highlight the association of ST‐segment elevation with SPM that may mimic acute pericarditis. This can pose a diagnostic challenge for clinicians and lead to unnecessary investigations and pharmacotherapy. Although the presentation can be dramatic, aggressive management is seldom required in these patients, even in the presence of a concurrent pneumopericardium without tamponade physiology.

## CONFLICT OF INTEREST

All authors declare no potential conflicts of interest to disclose related to the publication of this case series.

## AUTHOR CONTRIBUTIONS

HC and KS involved in conceptualization, patient consent, literature review, manuscript writing, data collection, and radiology part in writing and images. HC, KS, UN, and ZY involved in critical review and modifications. HC, KS, UN, TW, and ZY involved in final review and approval.

## CONSENT

Written informed consent was obtained from the patient for the publication of this case report and accompanying images.

## Data Availability

The data that support the findings of this study are available from the corresponding author upon reasonable request.
